# Complications after interval postpartum intrauterine device insertion

**DOI:** 10.1016/j.ajog.2021.08.028

**Published:** 2021-08-28

**Authors:** Mariana Ramos-Rivera, Sarah Averbach, Praveena Selvaduray, Amanda Gibson, Lynn L. Ngo

**Affiliations:** Division of Family Planning, Department of Obstetrics, Gynecology and Reproductive Sciences, University of California San Diego, San Diego, CA (Drs Ramos-Rivera and Averbach); One Medical, San Francisco, CA (Dr Selvaduray); Regional West Medical Center, Scottsbluff, NE (Dr Gibson); and Department of Obstetrics and Gynecology, Southern California Permanente Medical Group, San Diego, CA (Dr Ngo).

**Keywords:** interval intrauterine device insertion, intrauterine device expulsion, intrauterine device insertion complications, long-acting reversible contraception, postpartum, uterine perforation

## Abstract

**BACKGROUND:**

In the United States, up to 57% of women report resumption of sexual activity by the 6 week postpartum visit. Effective contraception should be addressed and provided at that time, to avoid unintended pregnancies and optimize interpregnancy intervals. Long-acting reversible contraceptives are the most effective forms of reversible contraception and are increasingly popular during the postpartum period. However, timing of postpartum intrauterine device (IUD) placement varies among providers and many delay insertion due to concerns for uterine perforation or expulsion of the IUD.

**OBJECTIVE:**

This study aimed to evaluate uterine perforation and expulsion rates with IUD insertion at 4–8 weeks postpartum vs 9–36 weeks postpartum.

**STUDY DESIGN:**

We performed a retrospective cohort study using the Kaiser Permanente Southern California electronic medical record from 2010 to 2016. We calculated the proportion of perforations and expulsions with IUD insertion at 4–8 weeks vs 9–36 weeks postpartum. Our primary outcome was the perforation rate. Secondarily, we evaluated the expulsion rate. For our minimum sample size calculation, to detect a difference of 0.5% in the perforation rate, with a baseline perforation rate of 0.5% for the 9–36 week postpartum IUD placement group, 80% power, and 5% alpha error rate, we would need at least 4221 participants per group, 8442 in total.

**RESULTS:**

A total of 24,959 patients met inclusion criteria (n=13,180 in the 4–8 week group, n=11,777 in the 9–36 week group). Of 430 patients with a confirmed complication, 157 uterine perforations and 273 IUD expulsions were identified. Perforation rates were significantly higher with placement at 4–8 weeks than at 9–36 weeks (0.78% vs 0.46%; *P*=.001). After adjusting for race and ethnicity, breastfeeding, IUD type, provider type, parity, most recent delivery, and body mass index, the odds of perforation remained higher with placement at 4–8 weeks than at 9–36 weeks (adjusted odds ratio, 1.92; 95% confidence interval, 1.28–2.89). Our Kaplan-Meier survival curve showed that the risk of uterine perforation remained elevated until approximately 22–23 weeks postpartum. Expulsion rates were similar between the 2 groups (1.02 vs 1.17; *P*=.52).

**CONCLUSION:**

Uterine perforation after interval postpartum IUD insertion is greater at 4–8 weeks than at 9–36 weeks, although perforation rates remain low at <1%. Expulsion rates did not differ between the groups. Because overall rates of uterine perforation are low, women can safely be offered IUDs at any interval beyond 4 weeks with minimal concern for perforation.

## Introduction

In the United States, 40% to 57% of women report sexual activity by the 6-week postpartum visit, putting them at risk for unintended pregnancy and short interpregnancy intervals.^1^ Unintended pregnancies are associated with increased risk of maternal depression and anxiety, and short interpregnancy intervals are associated with an increased risk of maternal and infant morbidity and mortality, including preterm birth, abruption, preterm premature rupture of membranes, and low birthweight.^2,3^ To optimize interpregnancy intervals and reduce unintended pregnancies, effective contraception should be addressed and provided in the postpartum period.^4^

Long-acting reversible contraception (LARC), which includes intrauterine devices (IUDs) and the etonogestrel subdermal contraceptive implant, are the most effective forms of reversible contraception. LARC use has steadily increased in the United States from about 2% in 2002 to 14% in 2014.^5^ LARC use is especially prevalent in the postpartum period, with use reported as 15% to 25% in the first 2–6 months postpartum.^5,6^ Moreover, women who choose IUDs and implants tend to have the highest contraceptive continuation rates.^7–9^

Timing of postpartum IUD placement varies among providers. Placement may be delayed because of concerns for uterine perforation at the postpartum visit. This results in additional clinic visits, which may reduce IUD uptake while increasing unintended pregnancies and overall costs. One large European prospective cohort study reported a 6-fold increased risk in perforation (6/1000) associated with breastfeeding for postpartum IUD insertions. However, a 2-fold risk persisted up to 36 weeks postpartum compared with a baseline risk of 1 in 1000.^10^ Overall, uterine perforation risk was low at <1% despite breastfeeding status. It is neither practical nor cost effective to delay IUD insertion to 36 weeks when risk returns to baseline.^11^ Moreover, interval IUD insertion at 4 weeks postpartum and beyond is category 1 (no restrictions) according to the Centers for Disease Control and Prevention (CDC) US Medical Eligibility Criteria for Contraceptive Use (MEC).^12^

Studies comparing the risk of postpartum IUD perforation were primarily performed in the 1980s and predominantly evaluated devices that are no longer available in the United States.^13,14^ The primary objective of this study is to compare uterine perforation rates with postpartum IUD insertion at 4–8 weeks postpartum vs 9–36 weeks postpartum. The secondary objective is to compare uterine expulsion rates for IUDs inserted at these postpartum intervals. We chose these specific intervals to capture the 6-week postpartum visit in the first interval and to include the earliest time that IUDs are considered category 1 by the CDC MEC.^13^ We hypothesized that the rate of uterine perforation at the time of postpartum IUD insertion would be greater in the 4–8 weeks postpartum period than the 9–36 weeks postpartum period but that the difference would not be clinically significant.

## Materials and Methods

We performed a retrospective cohort study with the Kaiser Permanente Southern California electronic medical record (EMR) from September 2010 to December 2016. Data collection started in 2010 when the specific diagnosis codes used to detect complications existed in the EMR. Data were extracted up to 2016 to allow for at least 1-year follow-up. This study was approved by the Kaiser Permanente Institutional Review Board. Inclusion criteria were the following: women who were at least 18 years old, had an IUD inserted between 4 and 36 weeks after delivery of an infant 24 weeks gestational age or greater and had follow-up for a year after placement. Patients were excluded if they delivered multiples or experienced uterine rupture. Patients identified by billing data as having a complication were manually reviewed to confirm the outcome diagnosis of uterine perforation or IUD expulsion. Because there were no specific International Classification of Diseases, Ninth or Tenth Revision (ICD 9 or 10) codes for uterine perforation caused by the IUD, we identified participants using ICD 9 and 10 codes for mechanical complication owing to intrauterine contraceptive device (996.32, T83.39), genitourinary complications owing to other implant and internal device (996.76), mechanical complication owing to other implant and internal device, (996.59) mechanical complication of genitourinary device (996.3), foreign body in uterus (939.1, T19.3), and displacement of intrauterine contraceptive device (T83.32). All data from manually reviewed patients were assessed for accuracy with double data entry.

Our primary outcome of uterine perforation was defined as laparoscopic-or imaging-confirmed perforation with any portion of the IUD noted beyond the endometrium or a provider-suspected perforation after sounding to a greater than expected depth. We subcategorized perforation by type as uterine (partial), uterine (complete), cervical, and provider-suspected perforation with sounding. Our secondary outcome of expulsion was subcategorized as partial or complete. Partial expulsions were defined as any part of the IUD noted within the cervix either visually on exam or on imaging.

In our manual chart review, we collected information on any pregnancy diagnosis, timing and setting of the IUD complication diagnosis, presenting symptom, and procedure to remove the IUD. We validated data that were ascertained from the EMR regarding weeks postpartum at the time of IUD placement, breastfeeding status, parity status (primiparous or multiparous, which was defined as 2 or more deliveries), type of most recent delivery, type of IUD, and provider type (attending, midlevel, or resident). General demographic data were collected for all patients, including age, body mass index (BMI), race/ethnicity, parity, number of vaginal deliveries, number of cesarean deliveries, most recent type of delivery, breastfeeding status, provider type, and type of IUD placed.

We calculated and compared the proportion of (1) perforations and (2) expulsions with postpartum IUD insertion at 4–8 weeks vs 9–36 weeks, diagnosed within 1 year of placement. The denominator to calculate this rate was the number of postpartum IUD insertions within the specified time frames. Secondarily, we analyzed the data using a multivariate logistic regression model to compare the odds of having a perforation and expulsion between the 2 groups to control for potential confounders. These potential confounders were race/ethnicity, breastfeeding status, IUD type, provider type, most recent delivery type, and BMI.^10^

We calculated our minimum sample size a priori, with the following assumptions. Given the reduced barrier for IUD placement if placed at the postpartum visit, we estimated that patients may still opt for IUD insertion at 4–8 weeks postpartum if the complication rate was low at 1% or less. Although IUD perforation is a rare event occurring at an overall incidence of 1.1–1.4 per 1000, it has been estimated to be higher in the postpartum breastfeeding population. In a recent prospective cohort study, the incidence was 5.6 per 1000 at ≤36 weeks postpartum (this decreases to 1.6 per 1000 at >36 weeks postpartum).^10^ For our minimum sample size calculation, to detect a difference of 0.5% in the IUD perforation rate, with a baseline complication rate of 0.5% for the 9 to 36 week postpartum IUD placement group, 80% power, and 5% alpha, we estimated we needed at least 4221 women per group (4–8 weeks and 9–36 weeks), for a total of 8442 women in total. The intervals of 4–8 and 9–36 weeks were chosen to capture the 6-week postpartum visit in the first interval, with a 2-week margin before and after 6 weeks.^13^

For our bivariate analyses, *P* values for comparing proportions were computed using chi-squared test or Fisher’s exact test, as appropriate based on cell size. Differences were considered statistically significant if *P*<.05. Any continuous outcomes were compared using *t* tests or Wilcoxon rank-sum tests, as appropriate based on normality of the data. We performed an unadjusted and adjusted multivariable regression model to determine the odds of a uterine perforation with IUD placement at 4–8 weeks compared with 9–36 weeks postpartum. The primary predictor was placement of the IUD at 4–8 weeks vs >9–36 weeks. We included covariates chosen a priori that have demonstrated clinical significance in the prior literature and/or are biologically plausible, which includes breastfeeding status, most recent delivery type, BMI (≤30 or ≥30), provider type, and IUD type.^10^ We added race/ethnicity to our regression model post-hoc owing to apparent differences between the 2 groups. An additional analysis was performed to look at the probability of IUD perforation through 36 weeks postpartum utilizing a Kaplan-Meier survival curve. All data were analyzed using Stata Statistical software: Release 15 (StataCorp LLC, College Station, TX).

## Results

A total of 24,959 patients met the inclusion criteria. We proceeded with this entire sample to optimize the power of our study to determine and compare the proportion of IUD perforations (a rare complication) and to allow for more variables to be included in our regression models. Of these patients, 841 were identified as having a potential complication. After manual review, 430 patients had the confirmed outcome diagnoses of uterine perforation or expulsion (51%).

There were 13,180 patients in the 4 to 8 week group and 11,777 patients in the 9–36 week group. Mean age, BMI, and IUD type were similar between the groups (Table 1). There was a higher proportion of cesarean deliveries in the 9–36 week group (22.9% vs 26.4%; *P*<.001). Breastfeeding status was higher in the 4–8 week group (59.3% vs 57.7%). There was also a higher proportion of Hispanic women in the 4–8 week group (60.3 % vs 53%; *P*<.001) and a higher proportion of White women in the 9 to 36 week group (21.2% vs 27.1%; *P*<.001).

Perforation was significantly higher with placement at 4–8 weeks postpartum than at 9–36 weeks postpartum (0.78% vs 0.46%; *P*=.001) (Table 2). Unadjusted expulsion rates were not significantly different between the 2 groups (1.02 vs 1.17; *P* = .52).

After controlling for race/ethnicity, breastfeeding status, IUD type, provider type, parity, most recent delivery type, and BMI (≤30 vs ≥30), the odds of any uterine perforation were significantly higher when IUDs were placed at 4–8 weeks than at 9–36 weeks postpartum (adjusted odds ratio [AOR], 1.92; 95% confidence interval [CI], 1.28–2.89; *P*=.002) (Table 3). Breastfeeding (AOR, 4.48; 95% CI, 1.95–10.33; *P*<.001), levonorgestrel IUD insertion (AOR, 1.84; 95% CI, 1.12–3.00; *P*=.02), multiparity (≥2 deliveries; AOR, 1.66; 95% CI, 1.09–2.52; *P* = .02), cesarean delivery (AOR, 1.68; 95% CI, 1.08–2.60; *P*=.02), and BMI ≥30 (AOR, 1.56; 95% CI, 1.04–2.34; *P*=.03) were all associated with significantly increased odds of perforation (Table 4). Race/ethnicity and provider type did not significantly affect the odds of perforation.

The AOR of IUD expulsion was similar in the 4–8 week group compared with the 9–36 week group (AOR, 0.98; 95% CI, 0.70–1.38; *P*=.92) (Table 3). Levonorgestrel IUD insertion (AOR, 0.61; 95% CI, 0.36–1.02; *P*<.001) and cesarean delivery (AOR, 0.59; 95% CI, 0.40–0.88; *P*=.01) were associated with decreased odds of expulsion (Table 4). Breastfeeding, race/ethnicity, multiparity (≥2 deliveries), provider type, and BMI ≥30 did not significantly affect the odds of expulsion.

A Kaplan-Meier survival curve was generated to evaluate the probability of IUD perforation through 36 weeks postpartum (Figure). It suggests a plateau in IUD perforation rates around 22–23 weeks after interval postpartum placement.

In our manual chart review of each complication, we collected further characteristics of those with complications. This included data on pregnancy diagnosis, timing and setting of the IUD complication diagnosis, presenting symptom, and procedure to remove the IUD (Table 5). There were 157 total perforations and 273 total expulsions. Pregnancy diagnosis in patients whose IUD was expelled was 5.9% (16/273) and in those with a uterine perforation was 2.5% (4/157). Most complications were identified at a clinic visit separate from the insertion visit (63.1% perforation and 92.2% expulsion). Of the patients who experienced uterine perforation, 35% (55/157) presented with pain, 12.1% (19/157) were unable to palpate their strings, and 36.3% (57/157) were asymptomatic. Of the patients whose IUD expelled, 30% (82/273) reported that their IUD had fallen out, 18.6% (51/273) presented for bleeding, 16.9% (46/273) were asymptomatic, and 16.5% (45/273) had pain. IUDs that perforated were removed laparoscopically in 64.6% (96/157) of cases, and 31.1% (46/157) were removed transvaginally. IUDs that expelled were only partially expulsed at the time of the complication diagnosis in 61.5% (168/273) of cases.

## Discussion

### Principle findings

Our study demonstrates a higher rate of uterine perforation with interval postpartum IUD placement at 4–8 weeks than at 9–36 weeks. The difference in perforation rate was 0.32%, less than our prespecified threshold for clinical significance, which was 0.5%. The rate of uterine perforation was low overall, at <1% in both groups. Breastfeeding status had the greatest impact on increasing the odds of uterine perforation. Our Kaplan-Meier survival curve illustrates the probability of uterine perforation with IUD placement plateauing around 22–23 weeks postpartum. Expulsion rates were not significantly different between insertion at 4–8 weeks (1.02%) and 9–36 weeks (1.17%) postpartum.

## Results

Two prospective studies from the 1980s evaluated the risk of IUD perforation based on timing of insertion postpartum and found no difference between the groups.^13,14^ Mishell et al^13^ assessed rates within 2 years following postpartum insertion of 5 different copper IUDs (only 1 is still used today in the United States) at either 4–8 weeks postpartum (n=411) or >8 weeks postpartum (n=1197) and showed no perforations. The study did not look at modern levonorgestrel IUDs. Heartwell et al^14^ conducted a case-control study to determine risk factors for uterine perforation and found no difference in perforation risk between insertions at <2 months vs >2 months.

The expulsion rates from our study were lower than expected, because previous studies reported rates between 2% and 30%, depending on the timing of insertion and length of follow-up.^15,16^ The lower rates in our study may be a result of coding errors, failure of patients to recognize and/or present to care after a complication, or seeking care outside of the Kaiser Permanente system (although we aimed to minimize this by including only patients with 1-year follow-up after placement with Kaiser). However, we do not expect these differences to be differential between our 2 study groups. Similar to our study, a different systematic review by Jatlaoui et al^15^ reported higher rates of expulsion after vaginal delivery (14.9%) than with cesarean delivery (3.6%).

### Clinical implications

Although the difference in perforation rates was statistically significant, the absolute difference was only 0.32%. This did not meet the prespecified power and sample size requirement for establishing what we considered clinically significant (≥0.5% difference). This is not unexpected given that our sample size was larger than our minimal amount needed to detect the 0.5% difference (4221 participants per arm were needed; n=13,180 in the 4–8 week group and n=11,777 in the 9–36 week group).

Moreover, our Kaplan-Meier survival curve illustrates the probability of uterine perforation with IUD placement plateauing around 22–23 weeks postpartum. The 22–23 week plateau is earlier than the 36 week plateau previously reported, possibly related to an earlier decline in breastfeeding in our population.^10^ We found that the odds of perforation were 4.5 times higher among those breastfeeding in the 4–8 week group than the 9–36 week group, similar to what was previously reported.^10^ Based on our data, providers would need to defer IUD insertion until at least 22 weeks postpartum to minimize the risk of perforation to baseline risk. This is neither practical nor advisable because most will have resumed sexual activity.^1,11^ With proper counseling, our data support offering interval postpartum IUD insertion any time at or beyond 4 weeks postpartum, without delay owing to a concern for perforation.

Expulsion rates did not differ between the 4–8 week and 9–36 week postpartum groups. Similar to the systematic review by Jatlaoui et al,^15^ cesarean delivery significantly decreased the risk of IUD expulsion. This is likely related to less overall cervical dilation in the cesarean group than the vaginal delivery group. In contrast to our study findings, Jatlaoui et al^15^ noted higher expulsion rates with the levonorgestrel IUD (15.5%) vs CuT380A IUD (10%) with an overall sample size of 58,000 participants. The differences in our study findings remain unclear. However, our lower odds of expulsion with the levonorgestrel IUD may be explained by the local effects of progesterone on the endometrial lining, possibly reducing bleeding and subsequently expulsion. Similar to Heinemann et al,^10^ our study also noted that perforation was more likely with the levonorgestrel IUD. This may be related to the differences between insertion devices.

### Research implications

Our study is limited by its retrospective design and reliance on accurate coding and documentation from many providers. Suggested future research includes prospective studies evaluating perforation and expulsion rates for immediate and interval postpartum IUD insertion. In addition, as the availability of postplacental IUD insertion increases, it would be useful to further explore other complications associated with the immediate postpartum period, such as postpartum hemorrhage and infection.

### Strengths and limitations

The strength of our study is the large sample size of a diverse group of patients, which increases generalizability and is ideal for evaluating rare events, such as IUD insertion complications. We were limited by the use of EMR documentation, which creates the possibility of bias because of coding by a variety of providers, and unmeasured confounding factors or missing data and results that may have been misclassified. Expulsion rates were slightly lower than expected, which may have been impacted by coding variability. However, all potential complications were manually reviewed and double data entry utilized to ensure accuracy.

### Conclusions

Although our study demonstrated higher perforation rates in the earlier interval postpartum placement group, the difference was not clinically significant and expulsion rates were not different. Given the significant positive public health impact of providing effective contraception soon after delivery, with proper counseling, patients should be offered IUD insertion at their desired postpartum time interval.

## Figures and Tables

**Figure:**
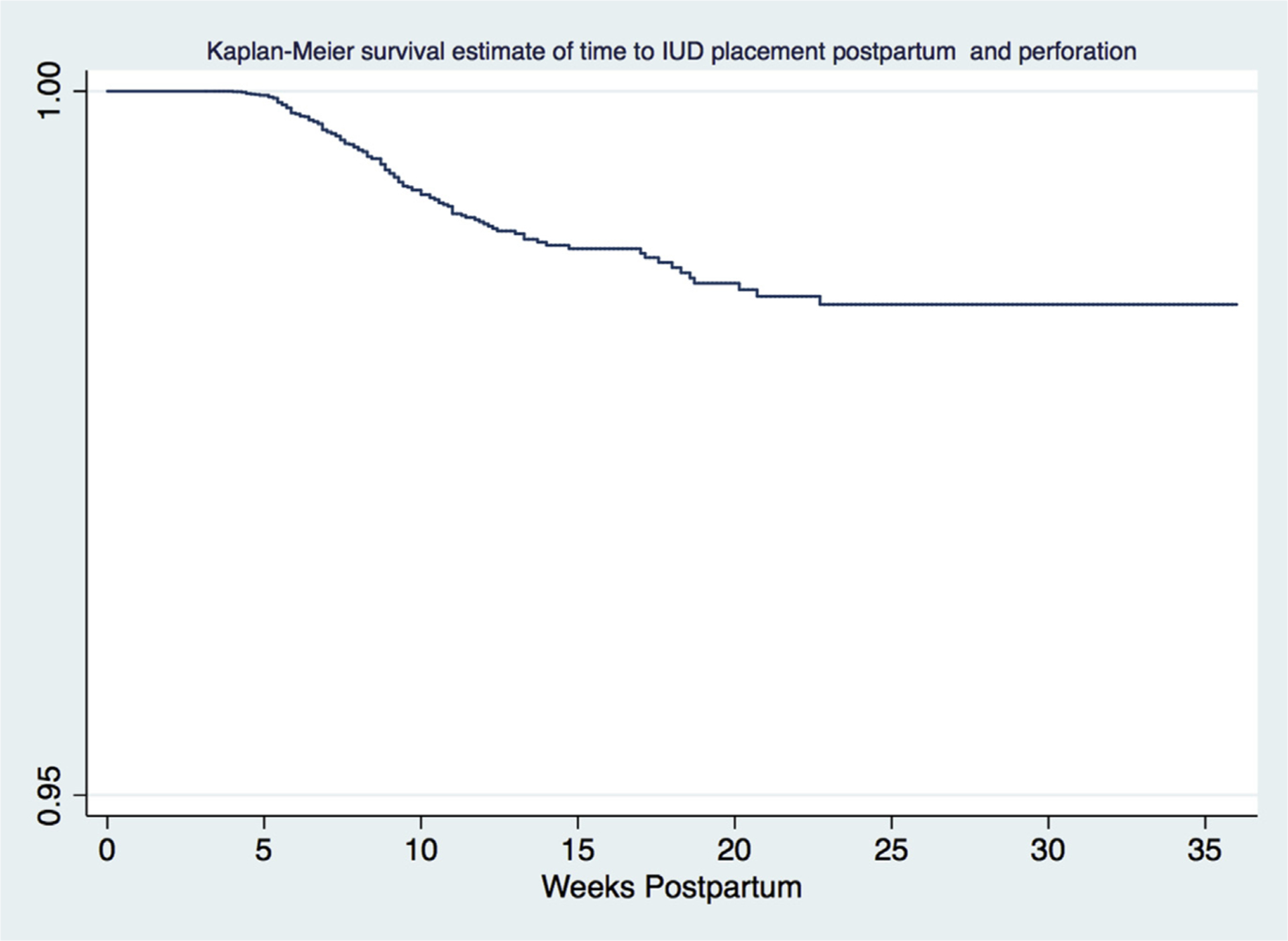
Kaplan-Meier survival estimate to time of IUD placement postpartum and perforation *IUD*, intrauterine device. Ramos-Rivera et al. Postpartum intrauterine device complications. Am J Obstet Gynecol 2022.

**TABLE 1:** Baseline characteristics

Patient characteristics	4–8 wk PP (n=13,180)	9–36 wk PP (n=11,777)	*P* value
Age (y)	28.8 (5.5)	29.1 (5.6)	<.001
BMI (kg/m^2^)	27.7 (9.1)	28.8 (9.6)	<.001
Race or ethnicity			<.001
White	2786 (21.1)	3190 (27.1)	
Black	1050 (8.0)	1058 (9.0)	
Hispanic	7945 (60.3)	6237 (53.0)	
Asian	1079 (8.2)	1058 (9.0)	
Multiple	299 (2.3)	355 (3.0)	
Most recent delivery			<.001
Vaginal	10,162 (77.1)	8670 (73.6)	
Cesarean	3018 (22.9)	3107 (26.4)	
Breastfeeding			<.001
Yes	5028 (59.3)	3996 (57.7)	
No	1944 (22.9)	2401 (34.7)	
Unknown	1510 (17.8)	531 (7.7)	
Provider type			.58
Attending physician	7000 (53.1)	5825 (49.5)	
APP (CNM, NP, PA)	5562 (42.2)	5426 (46.1)	
Resident physician	616 (4.7)	526 (4.5)	
IUD type			<.001
Copper IUD	3214 (24.4)	3019 (25.6)	
LNG-IUS	9966 (75.6)	8758 (74.4)	

Numeric variables are presented as mean (standard deviation) and categorical variables are presented as frequency (percentage).

*APP*, advanced practice provider; *BMI*, body mass index; *CNM*, certified nurse midwives; *IUD*, intrauterine devices; *LNG-IUS*, levonorgestrel releasing intrauterine system; *NP*, nurse practitioner; *PA*, physician assistant; *PP*, postpartum. *Ramos-Rivera et al. Postpartum intrauterine device complications. Am J Obstet Gynecol 2022*.

**TABLE 2:** Proportion of participants with complications by group

Complication	4–8 wk PP (n=13,180)	9–36 wk PP (n=11,777)	*P* value^a^
Perforation (any)	103 (0.78)	54 (0.46)	.001
Uterine, partial	31 (0.24)	16 (0.14)	.07
Uterine, complete	66 (0.50)	32 (0.27)	.004
Cervical	0 (0)	3 (0.03)	.07
With sounding	6 (0.05)	3 (0.03)	.41
Expulsion	135 (1.02)	138 (1.17)	.52
Partial	84 (0.64)	84 (0.71)	.46
Complete	51 (0.39)	54 (0.46)	.38

Data are presented as number (percentage).

*PP*, postpartum.

a *P* values are based on Pearson chi-square analysis.

Ramos-Rivera et al. Postpartum intrauterine device complications. Am J Obstet Gynecol 2022.

**TABLE 3:** Odds of perforation and expulsion among participants with postpartum IUD insertion at 4–8 weeks postpartum compared with 9–36 weeks postpartum

Placement at 4–8 wk PP	OR (95% CI)	*P* value	AOR (95% CI)	*P* value^‡a^
Perforation (any)	1.71 (1.22–2.38)	<.001	1.92 (1.28–2.89)	.002
Uterine, partial^b^	1.50 (0.89–2.55)	.13	1.66 (0.89–3.09)	.11
Uterine, complete	1.85 (1.21–2.82)	.004	1.84 (1.13–3.02)	.02
Expulsion	0.87 (0.69–1.11)	.26	0.98 (0.70–1.38)	.92
Partial	0.89 (0.66–1.21)	.46	1.13 (0.76–1.69)	.62
Complete	0.84 (0.57–1.24)	.38	0.82 (0.50–1.34)	.44

Values represented as number (percentage).

*AOR*, adjusted odds ratio; *CI*, confidence interval; *IUD*, intrauterine devices; *OR*, odds ratio; *PP*, postpartum.

a The study was not powered to evaluate expulsion rate,s subsets of perforation, or expulsion rates;

b Partial uterine perforation includes cervical perforation and suspected perforation with uterine sound.

Ramos-Rivera et al. Postpartum intrauterine device complications. Am J Obstet Gynecol 2022.

**TABLE 4:** Odds of any perforation or expulsion among women with postpartum intrauterine device insertion at 4–8 weeks postpartum compared with 9–36 weeks postpartum

	Perforation AOR (95% CI)	*P* value	Expulsion AOR (95% CI)	*P* value
Placement at 4–8 wk PP	1.92 (1.28–2.89)	.002	0.98 (0.70–1.37)	.92
Breastfeeding	4.48 (1.95–10.33)	<.001	0.61 (0.36–1.02)	.06
Levonorgestrel IUD	1.84 (1.12–3.00)	.02	0.49 (0.34–0.70)	<.001
Parity (≥2 deliveries)	1.66 (1.09–2.52)	.02	0.89 (0.63–1.25)	.50
Race or ethnicity^a^				
Black	0.31 (0.10–0.92)	.04	1.53 (0.68–3.42)	.30
Hispanic	0.54 (0.29–1.01)	.05	1.22 (0.66–2.22)	.53
Asian	0.77 (0.39–1.50)	.44	0.98 (0.50–1.89)	.95
Mixed, Other	1.39 (0.45–4.26)	.56	1.49 (0.48–4.63)	.50
Provider Type:				
APP^b^ (CNM, NP, PA)	1.34 (0.87–2.03)	.18	1.12 (0.79–1.58)	.45
Resident	1.92 (0.67–5.54)	.49	0.66 (0.20–2.11)	.44
Most recent delivery cesarean	1.68 (1.08–2.60)	.02	0.59 (0.40–0.88)	.01
BMI ≥30	1.56 (1.04–2.34)	.03	0.76 (0.53–1.07)	.12

Data are presented as number (percentage).

*AOR*, adjusted odds ratio; *APP*, advanced practice provider; *BMI*, body mass index; *CNM*, certified nurse midwives; *IUD*, intrauterine devices; *NP*, nurse practitioner; *PA*, physician assistant; *PP*, postpartum.

a Reference category for race or ethnicity is White, Non-Hispanic;

b Reference category for provider type is MD or DO.

Ramos-Rivera et al. Postpartum intrauterine device complications. Am J Obstet Gynecol 2022.

**TABLE 5:** Patient presentation characteristics for perforations and expulsions

Subcategory	Perforations (total n=157)					Expulsions (total n=273		
	Uterine, partial 47 (29.8)	Uterine, complete 98 (62.4)	Cervical 3 (1.9)	Provider-suspected perforation with sound 9 (5.7)	Total^a^ (%)	Partial 168 (61.5)	Complete 105 (38.5)	Total^a^ (%)
Pregnancy	0	4 (4.1)	0	0	4 (2.5)	13 (7.7)	3 (2.9)	16 (5.9)
Timing and setting of diagnosis								
- Clinic, time of IUD insertion	32 (68.1)	3 (3)	0	9 (100)	44 (28.0)	4 (2)	5 (4.8)	9 (3)
- Clinic, separate visit from insertion	10 (21.3)	86 (88)	3 (100)	0	99 (63.1)	154 (91)	97 (92.4)	251 (92)
- Emergency room or urgent care	5 (10.6)	9 (9)	0	0	14 (8.9)	9 (5)	3 (2.9)	12 (4)
Presenting symptom								
- Pain	8 (17.0)	46 (46.9)	1 (33)	0	55 (35.0)	40 (23.8)	5 (4.8)	45 (16.5)
- Bleeding	1 (2.1)	7 (7.1)	1 (33)	0	9 (5.7)	43 (25.6)	8 (7.6)	51 (18.6)
- Unable to palpate strings	2 (4.3)	17 (17.4)	0	0	19 (12.1)	2 (1.2)	4 (3.8)	6 (2.2)
- IUD fell out	0	0	0	0	0	0	82 (78.1)	82 (30.0)
- Asymptomatic, found at follow-up	4 (8.5)	24 (24.5)	1 (33)	0	29 (18.5)	38 22.6)	2 (1.9)	40 (14.7)
- Asymptomatic, found on immediate postplacement sono	27 (57.5)	1 (1.0)	0	0	28 (17.8)	4 (2.4)	2 (1.9)	6 (2.2)
- Patient or partner felt strings coming out or getting longer	1 (2.1)	0	0	0	1 (0.6)	12 (7.1)	0	12 (4.4)
- Other	4 (8.5)	3 (3.1)	0	9 (100)	16 (10.2)	29 (17.3)	2 (1.9)	31 (11.4)
IUD removal procedure								
- Transvaginal (standard)	41 (89)	3 (3.1)	2 (66.7)	0	46 (31.1)	160 (96.4)	7 (6.7)	167 (62)
- Hysteroscopic	3 (7)	0	0	0	3 (2.0)	2 (1.2)	0	2 (1)
- Laparoscopic	2 (4)	93 (94.9)	1(33.3)	0	96 (64.9)	0	0	0
- IUD fell out	0	0	0	0	0	0	98 (93.3)	98 (36)
- Other	0	2 (2.0)	0	0	2 (1.4)	4 (2.4)	0	4(2)

Percentage may not add up to 100 owing to rounding up of values.

*IUD*, intrauterine devices.

a Proportion of total perforations or expulsions.

Ramos-Rivera et al. Postpartum intrauterine device complications. Am J Obstet Gynecol 2022.

## AJOG at a Glance

### Why was this study conducted?

This study was conducted to evaluate the complication rates after intrauterine device insertion at 4 to 8 weeks vs 9 to 36 weeks postpartum.

### Key findings

The uterine perforation rate after interval postpartum intrauterine device insertion is greater at 4 to 8 weeks than at 9 to 36 weeks, although perforation rates remain low at <1% in both groups. Expulsion rates did not differ between the groups. Uterine perforation risk remains elevated above baseline until approximately 22–23 weeks postpartum.

### What does this add to what is known?

Because overall rates of uterine perforation are low, women can safely be offered intrauterine devices at any interval beyond 4 weeks postpartum with minimal concern for perforation.
